# Does Anti-TNF-α Therapy Affect the Bacteriological Profile of Specimens Collected from Perianal Lesions? A Retrospective Analysis in Patients with Crohn’s Disease

**DOI:** 10.3390/ijerph19052892

**Published:** 2022-03-02

**Authors:** Jolanta Gruszecka, Rafał Filip

**Affiliations:** 1 Institute of Health Sciences, Medical College of Rzeszow University, 35-310 Rzeszow, Poland; 2 Department of Clinical Microbiology, Clinical Hospital No. 2 im. Sw. Jadwigi Królowej, 35-301 Rzeszow, Poland; 3 Faculty of Medicine, Medical College of Rzeszow University, 35-959 Rzeszow, Poland; r.s.filip@wp.pl; 4 Department of Gastroenterology with IBD, Unit of Clinical Hospital 2 in Rzeszow, Lwowska 60, 35-301 Rzeszow, Poland

**Keywords:** Crohn’s disease, bacteriology of perianal abscesses, biological therapy

## Abstract

Anal abscesses and fistulas are potential complications of Crohn’s disease (CD). Chronic immunosuppression, loose stools, and poor wound healing in this population present challenges to the management of perianal diseases. The purpose of the study was to determine the predominant bacterial species found in specimens from perianal lesions causing anal abscess and/or fistula drainage in hospitalized patients, and to compare the number and type of microorganisms isolated from samples taken from patients undergoing biological therapy or traditionally treated. The outcomes of studies of patients treated for anal abscesses or fistulas from 2017 to 2019 were evaluated. Data obtained from medical records included culture and antibiotic sensitivity results of swabs from perianal lesions of isolated microorganisms. A total of 373 swabs were collected from perianal lesions during the analysis period, including 51 (49 positive samples) from patients with CD. The predominant pathogen was *Escherichia coli* (55%, *p* < 0.001), the second most common microorganism was *Staphylococcus aureus* (14.3%, *p* < 0.001). In vitro susceptibility testing showed *E. coli*, ESBL (strain with Extended Spectrum Beta-Lactamase) in five cases, *S. aureus*, MRSA (methicillin-resistant *S. aureus* -resistant to all beta-lactam antibiotics: penicillins with inhibitors, cephalosporins, monobactams, carbapenems, except for ceftaroline) in one sample. Biologic therapy does not affect the type of microorganisms isolated from perianal abscesses in patients with CD.

## 1. Introduction 

Both anal abscesses and fistulas are potential complications in the course of Crohn’s Disease (CD). Chronic immunosuppression, loose stools and poor wound healing in this population pose a challenge when treating the perianal disease [1,2]. It is commonly believed that the intestinal microbiome plays an essential role in the pathogenesis of Crohn’s disease, however, the microorganism or group of microorganisms involved remains elusive, despite technological advances in molecular biology that facilitate their detection. Using fecal samples and culture-independent techniques, several studies have reported that CD is associated with a decrease in Clostridiales, such as *Faecalibacterium prausnitzii* and an increase in Enterobacterales, such as *Escherichia coli* [3,4,5]. Nevertheless, it is important that the fistula tracts themselves lack mucosa-associated microbiota which may have relevance for the presumed microbial-immune interaction believed to drive inflammation [6].

The role of bacterial colonization in both pathogenesis of perianal abscesses and fistulas remains unclear at present; still, empirical antibiotic treatment, mostly comprising ciprofloxacin alone or in combination with metronidazole, is used on a regular basis [7,8]. Current data on microbial flora and specifically the resistance rates of bacteria found in perianal CD lesions are scarce. There are only single reports showing a non-pathological and most frequently polymicrobial growth pattern with a diversity of bacterial species. *Bacteroides*, *Escherichia coli, Enterococcus species*, coagulase-negative staphylococci (CoNS), *Staphylococcus aureus*, *Streptococcus viridans* and mixed anaerobic bacteria were the dominant types [8,9,10]. It is important here, since there are reports indicating that gram-negative aerobes isolated from abscesses in CD patients, more than two thirds are resistant to ciprofloxacin [11]. Having considered the foregoing, clinicians should consider this high rate of antibiotics resistance when choosing first-line antibiotic treatment for CD-related perianal lesions.

Since knowledge about resistance patterns is advantageous, in our study, we aimed to evaluate the microbial profile in a number of bacterial cultures obtained from perianal abscesses and fistula discharge.

## 2. Materials and Methods

### Ethics Statement

The study was approved by the Bioethics Committee of the Regional Medical Chamber (Resolution No. 88/B/2020 of 24 September 2020).

Pursuant to Polish law, patient consent was not required, due to the retrospective nature of the study.

In all patients studied, both perianal abscesses and fistulas were diagnosed on the basis of the MRI scan of the lesser pelvis. Radiological findings were evaluated by an experienced radiologist.

We analyzed the results of microbiological cultures from anal abscesses and fistula drainage in adult patients with Crohn’s disease admitted and subsequently treated between 1 January 2017 and 31 December 2019 at a tertiary IBD center in Rzeszow (southern Poland). Data of all hospitalized patients used for the analysis were obtained from the hospital’s electronic medical records. The material for the study was collected according to current procedures before starting the antibiotic therapy.

Samples were collected using sterile dry swabs tipped with a viscose swab, which were placed in tubes with Amies Transport Medium after the specimen was collected. The collected specimen was then inoculated onto solid media: Columbia agar with 5% sheep blood, MacConkey agar and Schaedler agar with 5% sheep blood. The media plates were incubated for 24–48 h at 37 °C under aerobic and anaerobic conditions. In case of growth on solid media, microorganisms were identified with a VITEK MS automated mass spectrometer (bioMérieux, Marcy-l’Étoile, France) using MALDI-TOF technology [12,13,14]. MS enables rapid, reliable identification of human pathogens and zoonotic and environmental microorganisms [15]. This technique, based on Matrix Assisted Laser Dessorption Ionization Time-of-Flight (MALDI-TOF), uses an extensive database of bacteria and fungi [16,17,18].

The drug resistance profile of cultured and identified microorganisms was determined by the disc diffusion method, or means of a VITEK2 (bioMérieux, France) automatic system for identification and determination of susceptibility, according to EUCAST (European Committee on Antimicrobial Susceptibility Testing) [19].

Statistical analysis was performed using PASW Statistics, version 18.0 from IBM (Armonk, New York, NY, USA).

## 3. Results

**Figure 1:**
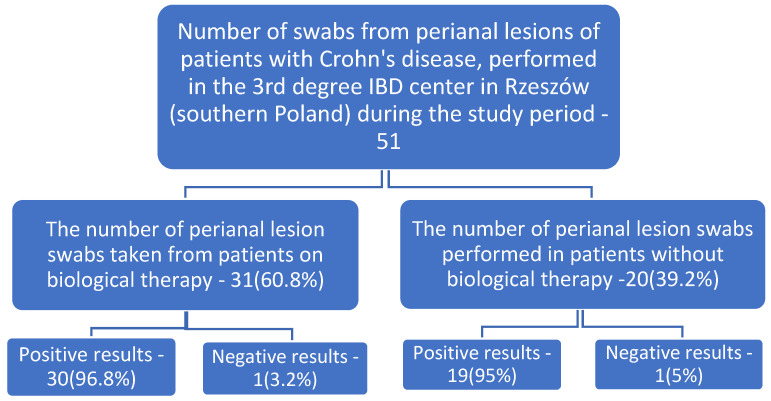
Percentages of positive and negative cultures in the population sampled.

Between January 2017 and December 2019, a total of 373 swabs from perianal lesions, including 51 from CD patients, were subjected to microbiological analysis. Microbial growth was found in 49 individuals with Crohn’s disease. Among the CD patients studied, the specimens for microbiological analysis were collected from 31 patients on biological therapy (among others anti-TNF-α) and 20 others—Figure 1.

The predominant aerobic bacteria in our study were: *Escherichia coli* (*n* = 27, of which *E. coli*, ESBL, *n* = 5), *Staphylococcus* microorganisms (*n* = 9, including *S. aureus*, *n* = 6, *S. aureus*, MRSA, *n* = 1, *S. epidermidis,* MRCNS, *n* = 2), *Enterococcus faecalis* (*n* = 5). The most abundant anaerobic isolate was *Bacteroides vulgatus* (*n* = 4). The frequency of other microorganisms is shown in Table 1.

Among patients on biologic therapy, bacterial growth was found in 30 samples (30/31—96.8%). The most frequently isolated microorganism was *Escherichia coli* (18/30—60%, *p* < 0.001), including *Escherichia coli,* ESBL from four swabs (strain with Extended Spectrum Beta-Lactamase). *Staphylococcus aureus,* was the second most abundant pathogen, being present in seven samples (7/30—23.3%, *p* < 0.001), including one *Staphylococcus aureus,* MRSA (methicillin-resistant *Staphylococcus aureus*—resistant to all beta-lactam antibiotics: penicillins with inhibitors, cephalosporins, monobactams, carbapenems, except for ceftaroline). This group of individuals was also diagnosed with e.g., *Staphylococcus epidermidis,* MRCNS in two samples (methicillin resistant coagulase-negative staphylococci—strain resistant to all beta-lactam antibiotics: penicillins, penicillins with B-lactamase inhibitor, cephalosporins and carbapenems)—Table 1. Among patients without biological therapy, microbial growth was noted in 19 samples (19/20—95%, *p* < 0.001). *Escherichia coli* was found most frequently (9/19—47.4%, *p* < 0.001), including *Escherichia coli,* ESBL in one sample. In vitro susceptibility testing showed *Klebsiella pneumoniae*, ESBL in two samples (2/19—10.5%). The results of culture of perianal lesion swabs from patients with Crohn’s disease without biological therapy are shown in Table 1.

Our study analyzed the microbiological findings of 49 adult patients with Crohn’s disease that showed microbial growth in specimens from perianal lesions. Out of 49 patients, 36 patients (73.5%, *p* < 0.001) were male and 13 patients (26.5%, *p* < 0.001) were female. The mean age of male and female patients was 38.9 ± 12.6 years (range, 21–65) and 29.9 ± 8 years (range, 18–50).

The analysis of the obtained results did not show any seasonal variation in the number of positive culture results of swabs from perianal lesions.

The Chi-square independence test confirmed the supposition that the type of therapy used in patients with Crohn’s disease did not affect the presence of microorganisms in perianal lesions.

Two groups of patients were included in the comparison: people undergoing biological therapy and without biological therapy, and the number of positive and negative test results in each group.

Characteristics of the patients with CD are presented in Table 2.

## 4. Discussion

In the three-year cohort of 373 adults analyzed, 51 perianal lesion swab cultures were performed on patients with Crohn’s disease, and 322 perianal lesion swab cultures were performed on other patients. Among the CD patients studied, 31 were on biologic therapy (e.g., anti-TNF-α) and 20 were without biologic therapy—Figure 1. The presence of pathogens in both groups of patients occurred with similar frequency: 96.8% vs. 95%. The predominant microorganism was *Escherichia coli* (including *E. coli,* ESBL)**,** which was diagnosed in 60% of specimens from patients on biological therapy and in 47.4% of specimens from patients without biological therapy—Table 1. 

In a study conducted in the city of Diyarbakir in south-eastern Turkey between January 2004 and December 2006, swabs from perianal abscesses taken from 81 patients, of whom 69 (86.4%) were male and 12 (13.6%) were female, were subjected to microbiological analysis. The mean ages of men and women were 40.5 ± 11.3 years (range, 21–67) and 35.8 ± 13 years (range, 16–51), respectively. Microorganism growth was found in 91.4% of samples (74/81). The dominant aerobic bacteria were: *Escherichia coli* (*n* = 36), coagulase-negative staphylococci (*n* = 16), *Enterococcus* spp. (*n* = 11) and *Staphylococcus aureus* (*n* = 10). Among the 10 *S. aureus* isolates, MRSA was responsible for 30%. The most common anaerobic pathogens were: *Bacteroides* spp. (*n* = 20) and *Peptostreptococcus* spp. (*n* = 6). The authors observed that aerobic organisms predominated in these infections [20]. 

In our present study, *Bacteroides vulgatus* was reported in 8.2% (4/49) of all positive test results. This type of bacteria, which are Gram-negative bacilli, belong to the absolute anaerobes. They are part of the physiological bacterial flora of the human gastrointestinal tract and predominate in abdominal infections and other infections that originate from the intestinal flora (i.e., perianal abscesses) [21].

The results of another prospective study conducted from September 2018 to July 2019 at the Central Hospital of Barquisimeto, Lara State, Venezuela, involving 42 patients diagnosed with anal abscesses were as follows: out all positive samples (34 abscesses), 21 (61.7%) had *Escherichia coli*, 10 (35.2%) samples contained *Klebsiella pneumoniae,* 2 (5.8%) positive samples showed the presence of *Proteus mirabilis* [22]. In all patients with fistulas, *E. coli* was isolated as the predominant microorganism. It is therefore considered to be a major predictor of anal fistulas [22].

The results of our study are similar to those previously reported. *Escherichia coli* was the most commonly identified microorganism in specimens collected from perianal lesions from patients with Crohn’s disease. It occurred in 55.1% (27/49) of positive samples, out of which *Escherichia coli,* ESBL was also diagnosed in five cases. The next most abundant pathogens were *Staphylococcus* bacteria, their presence was recorded in 18.4% (9/49) of positive samples: six (12.2%) *Staphylococcus aureus* isolates, one (2%) *Staphylococcus aureus*, MRSA isolate, two (4%) *Staphylococcus epidermidis,* MRCNS isolates—Table 1. 

Another study conducted in 2011 at Changi General Hospital, Singapore, involving 172 people, wherein specimens were collected from perianal abscesses for microbiological testing from 137 (112 positive) patients, gave the following results: 23 isolates were *Klebsiella* spp., 14 were *Escherichia coli*, two were *Actinomyces* spp., and 30 (26.8% of all positive results) belonged to the *Streptococcus* genus: 15 Group B *Streptococcus*, 12 *Streptococcus milleri*, two Group C *Streptococcus* and one *Streptococcus mitis*. Eight patients were diagnosed with *Staphylococcus aureus,* of which there were only two patients (1.5%) with multidrug-resistant *S*. *aureus* (MRSA). In this study, mixed enteric Gram-negative bacilli were found in 33 swabs from perianal abscesses [23].

In our present study, *Streptococcus* microorganisms accounted for 12.2% (6/49) of all positive microbiological findings. These included: *Streptococcus pyogenes*-2 isolates, *Streptococcus mitis*-2, *Streptococcus anginosus*-1, *Streptococcus constellatus*-1 isolate—Table 1. 

In a study conducted in Venezuela, two (5.8%) positive samples demonstrated the presence of *Proteus mirabilis* [22]. A recent review of consecutive Crohn’s disease patients with intra-abdominal abscesses as a result of active disease demonstrated *Proteus* spp. infection in 4.8% of cases [24]. 

In our present study, *Proteus mirabilis* was found in 6.1% (3/49) of all positive test results—Table 1. *Proteus* spp. are Gram-negative bacteria belonging to the *Enterobacteriaceae* family and are common commensal bacteria of the gastrointestinal microbiota [25]. The *Proteus* genus has been linked to postoperative recurrence of Crohn’s disease by two independent groups [26,27]. Studies of patients at the time of surgery, as well as 6 and 18 months after surgery showed that the microbiota comprising *Proteus* genus was detected in the majority of patients with relapse [26,27]. In a study by Mondot et al. involving 20 patients with Crohn’s disease undergoing ileocolonic resection, the presence of a *Proteus mirabilis* operational taxonomic unit (OTU) was predictive of recurrence at 6 months after the surgery [26]. 

The association in both studies was established prospectively and longitudinally, with predictive association, making a pathogenic role more likely [28].

*Proteus* bacteria can colonize medical devices placed in the gastrointestinal tract, including ventriculo-peritoneal shunts [29], nasogastric probes [30,31], biliary probes, and pancreatic stents [32], and tracheoesophageal voice prostheses [33]. It has been shown that *Proteus* bacteria can contaminate gastroscopes and colonoscopes in cases of inadequate, short-time disinfection [34]. Infection can also start in the hospital settings due to environmental contamination; *P. vulgaris* persists on dry, hard surfaces for up to 2 days [35]. There are reports of nosocomial and community outbreaks associated with person-to-person transmission of infection, with most patients becoming gastrointestinal carriers before infection [36]. There may be a link between *Proteus* bacteria and inflammatory bowel disease, specifically the Crohn’s disease [28]. 

The aim of our hospital-based study was to determine the predominant bacterial species present in specimens collected from perianal abscesses and fistula secretions in hospitalized patients of one of the IBD tertiary centers in Poland. 

The most abundant microorganism was *Escherichia coli* found in 55.1% (27/49) of all positive microbiological test results. The next most commonly isolated pathogens were *Staphylococcus* bacteria present in 18.4% (9/49) of positive results (including *S. aureus* 6/49—12.2%, *S. aureus*, MRSA 1/49—2%, *Staphylococcus* MRCNS 2/49—4.1%), *Enterococcus faecalis* present in 10.2% (5/49) of positive results, *Bacteroides vulgatus* in 8.2% (4/49) of positive results—Table 1. 

The second objective of our study was to compare the number and the type of microorganisms isolated from samples taken from patients on biologic therapy (among others anti-TNF-α) and those treated without biologic therapy. In both groups, the predominant pathogen was *Escherichia coli* found in 60% (18/30) of all positives among those on biologic therapy, including 4 (13.3%) isolates of *E. coli,* ESBL. Results of microbiological analysis of specimens from patients without biological therapy showed that *Escherichia coli* was present in 47.4% (9/19) of all positive results, including one sample of *E. coli,* ESBL (5.3%). *Staphylococcus* bacteria were also diagnosed in the group of patients on biological therapy in 9 cases (*Staphylococcus aureus*-6/30, 20%, *Staphylococcus,* MRSA-1/30, 3.3%, *Staphylococcus,* MRCNS-2/30, 6.7%). No staphylococci were found among patients without biologic therapy. In this group of patients, *Bacteroides vulgatus* was isolated in 15.8% (3/19) of all positive samples, while *Bacteroides vulgatus* was isolated in 3.3% (1/30) of all positive results in the group of patients undergoing biological therapy—Table 1.

In contrast to our findings, the literature on adults has reported a predominance of Gram-positive bacteria, particularly staphylococci and streptococci, over Gram-negative intestinal organisms in swabs collected from perianal lesions [37]. It was found that in Crohn’s disease, perianal fistulas are predominantly colonized by Gram-positive microorganisms. Having considered the foregoing, antimicrobial treatment of this condition should target such microorganisms [37]. 

Perianal abscesses are more common in men than in women [38,39]. Our study also showed that positive culture of swabs from anal lesions was significantly more common in samples from men (36 samples—73.5%) than from women (13 samples—26.5%); the male-to-female ratio was 2.8. In a retrospective study conducted from January 2004 to December 2006 at the University Hospital in the city of Diyarbakir in southeastern Turkey, the male-to-female ratio was 5.7, similar to the study by Lunniss and Philips [40]. In the pediatric population, the male predominance is even more pronounced [41]. Other similar studies have also found that perianal abscesses are more common in men than in women [20,22,23,38]. Our results are consistent with them.

The peak incidence of anal and rectal abscesses occurs in the third and fourth decades of life [20,22,23,38]. The average age of patients is around 40 years [20,23,38,42,43,44]. In England, for example, most cases occur around the age of 40, with an annual incidence of 19/100,000 [45]. In the United States, similarly, most cases of perianal abscesses are reported in the third or fourth decade of life, with annual incidence ranging from 70 to 150 cases per 100,000 people [1]. 

In our study, the mean age of male and female patients was 38.9 ± 12.6 years (range, 21–65) and 29.9 ± 8 years (range, 18–50), respectively. Risk factors associated with the development of anal abscesses include obesity, diabetes, sedentary lifestyle, smoking, and previous rectal surgery [46]. To this day, it is unclear whether changes in the intestinal microflora in inflammatory bowel disease are a cause of the disease, a consequence of the disease, or unrelated to the disease [47].

Literature includes opinions on the low usefulness of microbiological test results based on specimens collected from perianal lesions, when used in therapeutic management. In some hospitals, all patients were discharged before microbiological analysis results were available [48]. Although the cost of testing is not high, complete microbiologic analysis of each culture, including susceptibility testing, can be labor-intensive [49]. Because of that, some authors do not recommend the routine collection of perianal abscess swabs unless there are clinical problems, such as: recurrent perianal sepsis, immunocompromised status, or extensive soft tissue necrosis, especially when these features are associated with systemic sepsis [48]. 

In contrast, other authors believe that treatment of perianal abscesses requires prompt surgical drainage and antimicrobial therapy [37]. All infections occurring in perianal lesions can be life-threatening for patients who are immunocompromised or suffer from a malignant neoplastic disease [50]. Because of that, it is important to identify the causative organisms; this is why access to microbiologic analysis is necessary [37]. 

The gut microbiota is a crucial environmental factor in the development of multifactorial diseases, such as obesity, diabetes, rheumatoid arthritis, and inflammatory bowel diseases represented by Crohn’s disease and ulcerative colitis. As the link between various diseases and aberrant intestinal microbiota becomes apparent, there is an urgent need to develop and disseminate control strategies for dysbiosis in addition to existing effective treatments [51].

## 5. Conclusions

The use of biologic therapy has no effect on the type of microflora isolated from perianal abscesses in patients with Crohn’s disease. Our results are generally not different from those of microbiological analyses in other countries. 

The role of the gut microbiome and/or dysbiosis in the etiology of perianal lesions in CD is not fully elucidated yet. For CD patients, any bacterial infection poses a serious health risk, during especially immunosuppressive and/or biological therapy. Clinical practice shows that apart from surgical treatment, long-term antibiotic therapy brings very good results. Microbiological tests are important in the absence of any effects of empiric therapy or the emergence of septic complications.

The study presented here has some limitations. This is a retrospective study with a small number of patients, therefore further prospective studies with larger numbers of patients are needed.

## Figures and Tables

**Table 1:** Results of studies on cultures of perianal lesions in patients in a tertiary center in Rzeszow (southern Poland) (January 2017–December 2019).

Number of Tests Ordered *n*	Positive Results *n* (%)	Microorganisms Cultured	Number	% in Relation to All Samples Taken	% in Relation to Positive Results	Significance Level *p*
**Summary results**						
51	49 (96%)	*Escherichia coli*	22	43.13%	44.9%	<0.001
		*Escherichia coli,* ESBL	5	9.8%	10.2%	<0.001
		*Staphylococcus aureus*	6	11.8%	12.2%	<0.001
		*Staphylococcus aureus,* MRSA	1	1.96%	2.%	=0.322
		*Enterococcus faecalis*	5	9.8%	10.2%	<0.001
		*Bacteroides vulgatus*	4	7.8%	8.2%	<0.001
		*Proteus mirabilis*	3	5.9%	6.1%	=0.004
		*Staphylococcus epidermidis,* MRCNS	2	3.9%	4.1%	=0.051
		*Enterobacter cloacae*	2	3.9%	4.1%	=0.051
		*Streptococcus pyogenes*	2	3.9%	4.1%	=0.051
		*Klebsiella pneumoniae,* ESBL	2	3.9%	4.1%	=0.051
		*Klebsiella pneumoniae*	2	3.9%	4.1%	=0.051
		*Streptococcus mitis*	2	3.9%	4.1%	=0.051
		*Pseudomonas aeruginosa*	1	1.96%	2%	=0.322
		*Morganella morganii*	1	1.96%	2%	=0.322
		*Citrobacter freundii*	1	1.96%	2%	=0.322
		*Streptococcus anginosus*	1	1.96%	2%	=0.322
		*Prevotella disiens*	1	1.96%	2%	=0.322
		*Parvimonas micra*	1	1.96%	2%	=0.322
		*Streptococcus constellatus*	1	1.96%	2%	=0.322
**Results of tests performed on patients during biological therapy**						
31	30 (96.8%)	*Escherichia coli*	14	45.2%	46.7%	<0.001
		*Escherichia coli,* ESBL	4	12.9%	13.3%	<0.001
		*Staphylococcus aureus*	6	19.35%	20%	<0.001
		*Staphylococcus aureus,* MRSA	1	3.2%	3.3%	=0.325
		*Enterococcus faecalis*	3	9.7%	10%	=0.005
		*Proteus mirabilis*	2	6.4%	6.7%	=0.055
		*Staphylococcus epidermidis,* MRCNS	2	6.4%	6.7%	=0.055
		*Klebsiella pneumoniae*	2	6.4%	6.7%	=0.055
		*Bacteroides vulgatus*	1	3.2%	3.3%	=0.325
		*Enterobacter cloacae*	1	3.2%	3.3%	=0.325
		*Streptococcus mitis*	1	3.2%	3.3%	=0.325
		*Morganella morganii*	1	3.2%	3.3%	=0.325
		*Citrobacter freundii*	1	3.2%	3.3%	=0.325
		*Prevotella disiens*	1	3.2%	3.3%	=0.325
		*Parvimonas micra*	1	3.2%	3.3%	=0.325
**Results of tests performed on patients without biological therapy**						
20	19 (95%)	*Escherichia coli*	8	40%	42.1%	<0.001
		*Escherichia coli,* ESBL	1	5%	5.3%	=0.329
		*Bacteroides vulgatus*	3	15%	15.8%	<0.007
		*Enterococcus faecalis*	2	10%	10.5%	=0.059
		*Streptococcus pyogenes*	2	10%	10.5%	=0.059
		*Klebsiella pneumoniae,* ESBL	2	10%	10.5%	=0.059
		*Proteus mirabilis*	1	5%	5.3%	=0.329
		*Enterobacter cloacae*	1	5%	5.3%	=0.329
		*Streptococcus mitis*	1	5%	5.3%	=0.329
		*Pseudomonas aeruginosa*	1	5%	5.3%	=0.329
		*Streptococcus anginosus*	1	5%	5.3%	=0.329
		*Streptococcus constellatus*	1	5%	5.3%	=0.329

MRCNS—methicillin-resistant coagulase-negative staphylococci (strain resistant to all beta-lactam antibiotics: penicillins, penicillins with B-lactamase inhibitor, cephalosporins and carbapenems). MRSA—methicillin-resistant *Staphylococcus aureus* (resistant to all beta-lactam antibiotics: penicillins with inhibitors, cephalosporins, monobactams, carbapenems, except for ceftaroline). ESBL—strain with Extended Spectrum Beta-Lactamase.

**Table 2:** Characteristics of the patients with CD.

Characteristics of the Patient	CD (*n* = 51)			
	Patients during Biological Therapy		Patients without Biological Therapy	
	Women (*n* = 6)	Men (*n* = 25)	Women (*n* = 9)	Men (*n* = 11)
Age, years	18–33	24–57	26–50	2165
Age, mean (standard deviation)	24.33 (7.203)	37.95 (9)	34.667 (8.994)	39.412 (15.069)
Length of hospital stay, days	2–35			
Length of hospital stay, mean (standard deviation)	11.6 (7.1)			
Onset of symptoms prior to admission to hospital, weeks	1–8			
Onset of symptoms prior to admission to hospital, mean (standard deviation)	3.863 (6.103)			
Taking samples for research	All samples were taken during hospitalization			

## Data Availability

The data underlying this article will be shared on reasonable request to the corresponding author.
